# RT-ring: a small wearable device for tremulous Parkinson’s disease diagnosis in primary care

**DOI:** 10.3389/fneur.2025.1534205

**Published:** 2025-01-27

**Authors:** Jolanda Buonocore, Basilio Vescio, Marida De Maria, Marianna Crasà, Rita Nisticò, Pier P. Arcuri, Giuseppe L. Cascini, Anna Latorre, Aldo Quattrone, Andrea Quattrone

**Affiliations:** ^1^Institute of Neurology, Department of Medical and Surgical Sciences, Magna Graecia University, Catanzaro, Italy; ^2^Neuroscience Research Center, Magna Graecia University, Catanzaro, Italy; ^3^Biotecnomed S.c.ar.l., Catanzaro, Italy; ^4^Institute of Bioimaging and Complex Biological Systems, IBSBC-CNR, Catanzaro, Italy; ^5^Institute of Radiology, Azienda Ospedaliero-Universitaria Renato Dulbecco, Catanzaro, Italy; ^6^Nuclear Medicine Unit, Azienda Ospedaliera-Universitaria Dulbecco, Catanzaro, Italy; ^7^Department of Experimental and Clinical Medicine, Magna Graecia University, Catanzaro, Italy; ^8^Department of Clinical and Movement Neurosciences, UCL Queen Square Institute of Neurology, University College London, London, United Kingdom

**Keywords:** rest tremor, tremor pattern, wearable device, RT-ring, DaTscan, Parkinson’s disease, essential tremor plus, machine learning

## Abstract

**Introduction:**

Differential diagnosis of rest tremor (RT) disorders is challenging, often requiring 123I-ioflupane single-photon-emission-computed tomography (DaTscan), an expensive technique not available worldwide. In the current study, we investigated the performance of a new wearable mobile device termed “RT-ring” in predicting DaTscan result in patients presenting with RT based on rest tremor inertial features.

**Methods:**

Consecutive RT patients underwent RT-ring tremor analysis, surface electromyography (sEMG), and DaTscan. The RT-ring is a miniaturized mobile device that uses machine learning based on inertial tremor data to estimate the RT pattern. This electrophysiologic tremor feature has proven to accurately predict DaTscan result. The primary outcome was the RT-ring’s performance in distinguishing patients with and without striatal dopaminergic deficit.

**Results:**

Sixty-seven RT patients were enrolled, including 42 patients with striatal dopaminergic deficit and 25 with normal DaTscan. The RT-ring showed 85.0% sensitivity, 90.9% specificity, and 87.9% balanced accuracy in predicting DaTscan result, and demonstrated 96.8% agreement with sEMG in RT pattern classification.

**Conclusion:**

The RT-ring is a promising, non-invasive, user-friendly, wearable mobile device for supporting the diagnosis of tremulous Parkinson’s disease in primary care settings, especially in low-income countries with limited access to dopamine imaging.

## 1 Introduction

Upper limb rest tremor (RT) is a typical presenting symptom of Parkinson’s disease (PD); however, it can be observed in essential tremor (ET) plus, dystonic or drug-induced tremor (1–4). The DaTscan (123I-ioflupane) often guides the differential diagnosis (1, 5, 6) but is unsuitable for routine use due to high costs, invasiveness long waiting lists in high-income countries, or limited availability in low-income countries with resource-constrained settings and rural areas. Therefore, simple biomarkers are needed to support routine clinical RT diagnosis and select patients for second-level diagnostic procedures.

Previous studies demonstrated that the RT muscular contraction pattern evaluation through surface electromyography (sEMG) can differentiate Parkinsonian tremor (alternating pattern) from other RT syndromes with no damage of the dopaminergic system (typically showing a synchronous pattern) (7–15). Unlike postural tremor, often showing variability of tremor features over time with spontaneous shifts across alternating and synchronous patterns (13, 16), rest tremor shows a very stable pattern over time, also across multiple recordings (10–13). We have recently developed a small, user-friendly, ring-shaped wearable device termed “RT-ring,” which, by being worn on a finger of the tremulous hand, can estimate the RT pattern, a tremor characteristic that has proven to accurately predict DaTscan result (10). RT-Ring employed robust machine learning technology based on tremor inertial data (17), overcoming the main limitations of sEMG (expertise and subjective assessment). The objective of the current study was to investigate the RT-ring performance in distinguishing RT patients with or without striatal dopaminergic deficit (using the DaTscan as ground truth), establishing the role of this portable and user-friendly device as surrogate biomarker of dopamine imaging in RT patients for routine clinical practice.

## 2 Methods

### 2.1 Patients

Patients presenting with asymmetric upper limb RT were consecutively enrolled at the Institute of Neurology and Neuroscience Research Center of the Magna Graecia University of Catanzaro between January 2023 and May 2024. The study protocol included a neurological examination performed by movement disorder specialists, brain 3 T-MRI, tremor analysis using sEMG and “RT-ring,” followed by DaTscan within 3 months. To ensure accurate tremor assessment, all medications known to interfere with tremor were suspended at least 2 days before the examination. Patients on dopaminergic therapy were evaluated in the ‘OFF’ state (off medications overnight). Exclusion criteria were the presence of prominent bradykinesia or rigidity [defined as score > 2 on items 3.3-to-3.6 of the Movement-Disorders-Society-Unified-Parkinson’s-Disease-Rating-Scale pars-III (MDS-UPDRS-III)] (18), and widespread vascular lesions or neoplasia on MRI. The institutional review board (Magna Graecia University review board, Catanzaro, Italy) approved all study procedures and ethical aspects. All study participants gave written informed consent.

### 2.2 Tremor analysis

#### 2.2.1 RT-ring

**Figure 1:**
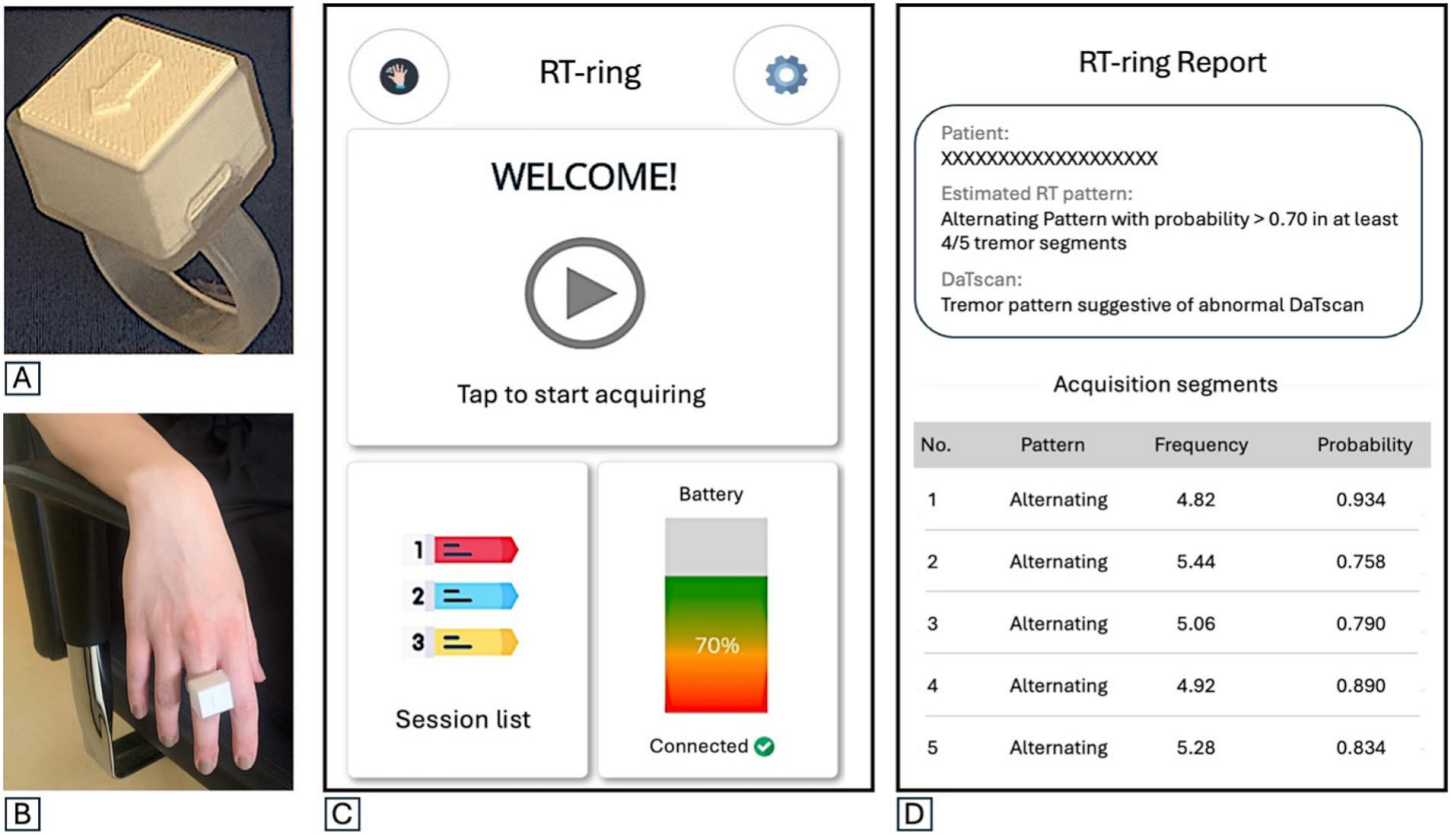
**(A)** The RT-ring device. **(B)** The RT-ring worn on a subject’s finger. **(C)** The mobile app’s initial screen shows three boxes: one to start a new session, one to see and access the session list with previous recordings, and one showing the device battery charge level and the connection status between the app and the device. **(D)** An example of the RT-ring report with the patient’s name or identifier, the estimated predominant rest tremor pattern, the prediction on DaTscan result, and a summary of the characteristics for each of the five tremor recording segments (frequency, estimated pattern and probability of pattern estimation). In the report shown in part label **D**, in the RT-ring report, frequency is provided in Hz.

**Figure 2:**
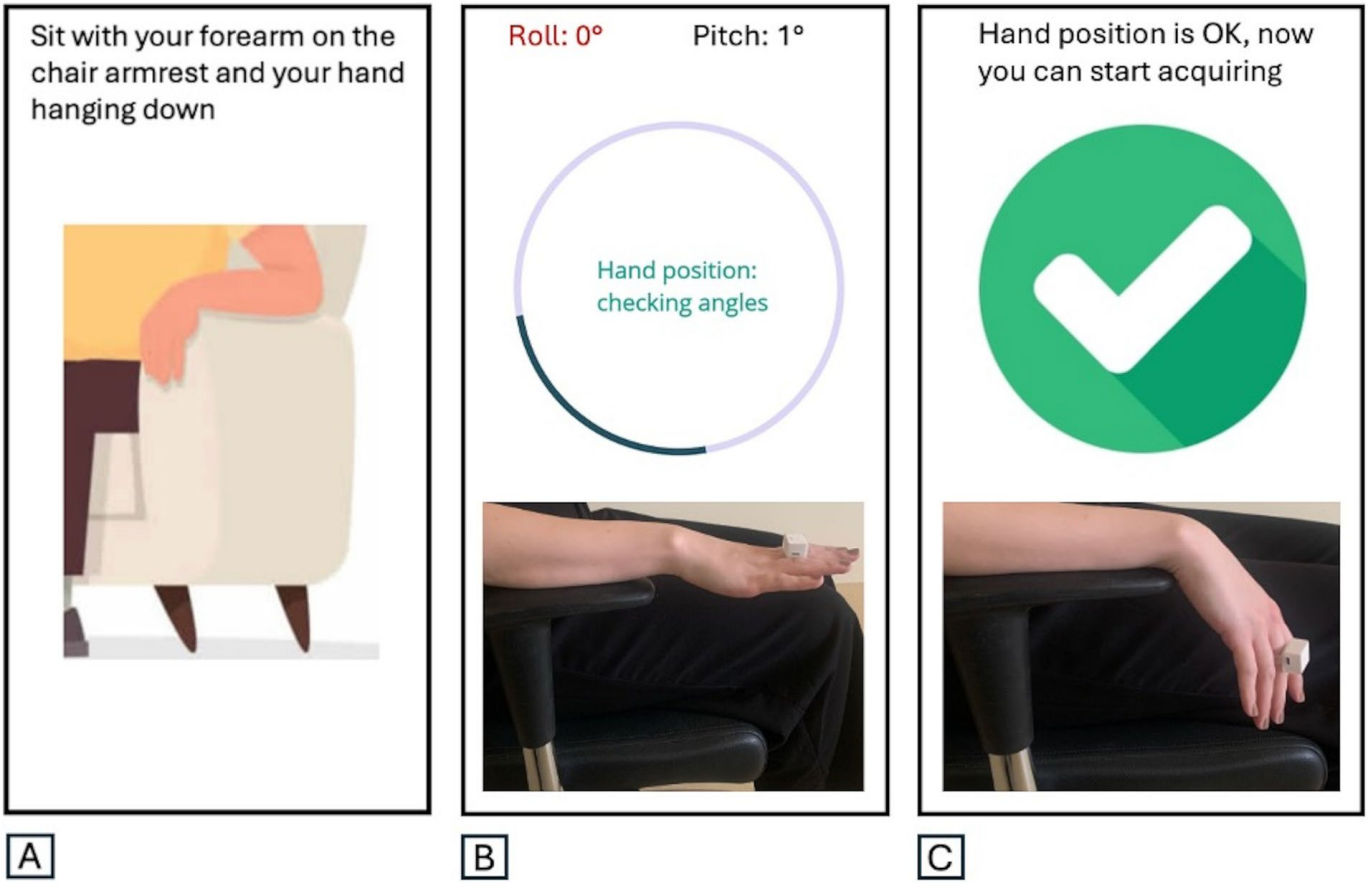
The correct hand positioning is checked through tilt sensors (roll and pitch angle evaluation). **(A)** The correct positioning of the hand for rest tremor recording is shown on the graphic user interface. **(B)** Incorrectly positioned hand: roll in red. **(C)** Correctly positioned hand: recording can be started.

**Figure 3:**
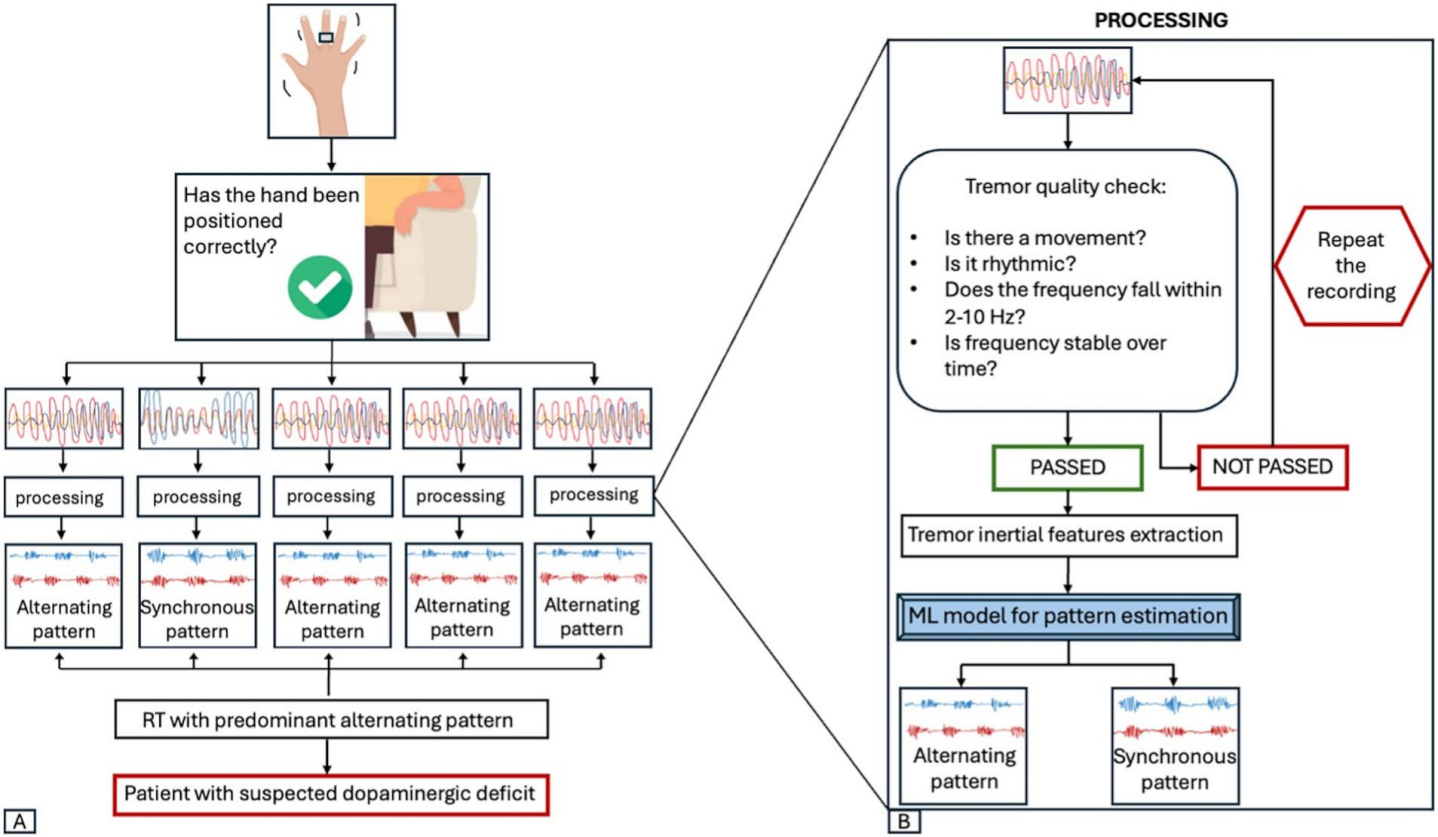
RT-ring workflow. **(A)** When starting a new session, the correct positioning of the hand is checked. The recording begins only when the hand is hanging down from the chair armrest. The app collects five 10-s segments, each undergoing a quality control process. The tremor pattern of each segment is then estimated using a machine-learning (ML) model based on tremor inertial features. If the same pattern (alternating or synchronous) is estimated with a probability >70% by the ML model in at least 4 out of 5 segments, the patient’s tremor is classified as predominantly alternating or synchronous and the prediction on the DaTscan result is shown. **(B)** The processing of a single 10-s recording is shown. Each segment undergoes a tremor quality check (QC) to ensure there is a rhythmic hand movement with a frequency between 2 and 10 Hz, without significant frequency shifts over time. If a segment does not meet these criteria, it is discarded and a new segment is recorded, until 5 segments passing the QC are collected. Tremor inertial features are then extracted from each recording segment and used as input for a ML model to predict the tremor pattern of the RT segment. RT, rest tremor; ML, machine-learning.

The RT-ring is a miniaturized wearable device consisting of a small hardware with a printed circuit board and a rechargeable battery, mounted on a ring-shape silicon support to be worn on a finger (Figures 1A,B). Technical details on the hardware structure are provided in a previous publication (17). Tremor inertial data are collected using a triaxial accelerometer and gyroscope and transmitted to a smartphone app via Bluetooth Low Energy. The mobile app has a user-friendly graphic user interface (GUI) designed to guide the physician through the tremor recording procedure, providing step-by-step instructions that enable its wide use by people with no experience in the field. The app home screen includes one box to start a new session, one to access the previous sessions, and one showing the device battery level and connection status (Figure 1C). Several quality checks were implemented to ensure reliable data acquisition, as shown in Supplementary Figures S1, S2. First, the correct hand positioning is checked through tilt sensors, aiming to minimize the possibility of postural tremor; the recording begins when the hand hangs from the chair armrest, as shown in Figure 2. Five 10-s tremor recording segments are acquired, and each segment undergoes a comprehensive quality control (QC) process to confirm the presence of tremor, defined as a rhythmic movement with a stable frequency falling between 2 and 10 Hz (Figure 3), as typically observed in most hand tremor disorders (2); recording segments not passing QC are discarded. Inertial tremor features are extracted from tremor segments and used as input for a random forest model to estimate the RT pattern without sEMG. The model building was previously described (17). It was trained and tested on a dataset of 389 RT segments from an independent patient cohort with 70–30 splitting procedure, showing accuracy: 0.98 (0.93–1.00) in estimating the pattern of RT segments, and it was frozen before the current study. If at least 4/5 segments show a consistent pattern, each with high prediction probability (≥70%), the patient’s tremor is classified as predominantly alternating or synchronous (Supplementary Figure S1A); otherwise, the RT-ring recording session is repeated. A report is then generated, including the estimated tremor pattern, the DaTscan result prediction, and a summary of the characteristics of the acquired tremor segments, as shown in Figure 1D.

#### 2.2.2 Surface electromyography

All patients also underwent sEMG electrophysiological analysis of RT in the most affected upper limb, contemporary to the RT-ring assessment for evaluating RT-ring accuracy in pattern estimation compared to the gold standard sEMG technique. The rest tremor activity was recorded by surface electrodes from antagonistic forearm muscles (extensor carpi radialis and flexor carpi ulnaris muscles), using an Electromyograph Dantec Keypoint system by Natus Neurology, as previously described (7–10). All drugs that might interfere with tremor were suspended 2 days before the examination. The patient was seated in a comfortable chair with the arm flexed at 90 degrees, the forearm fully supported against gravity, and the hand hanging down from the armrest. The tremor assessment was performed at rest without any motor task. A standardized cognitive task (subtracting 3 or 7 by a hundred) was employed before the recording started to bring out the RT in patients with slight and intermittent tremor. Five recording segments, each characterized by 10 s with rest tremor, were considered for each patient. The bursts were manually segmented from the filtered sEMG signals, and the mean burst amplitude was evaluated. Spectral analysis was performed to extract the frequency associated with the tremor peak. The contraction pattern of antagonistic muscles was visually assessed on sEMG recordings by two independent raters (a neurologist and a technician expert in tremor analysis) blinded to the clinical diagnoses, and it was classified as alternating or synchronous; a third rater evaluated the traces in case of discrepancy. The tremor pattern reflects the temporal relationship between contraction bursts in antagonist muscles. The term “synchronous” was used to describe a pattern characterized by intermittent contractions of the antagonist muscles occurring simultaneously, while the term “alternating” reflected a temporal shift between the two muscles’ contractions (11, 19, 20).

### 2.3 MRI acquisition protocol

All MRI scans were performed with the same 3-T MR750 General Electric scanner with an 8-channel head coil (Discovery MR- 750, GE, Milwaukee, WI, United States). The acquisition protocol included: a 3-dimensional T1-weighted volumetric spoiled gradient echo (GE) (sagittal section; repetition time/echo time 9.2/3.7 milliseconds; slice thickness 1.0 mm; frequency and phase encoding matrix 256 × 256; flip angle 12°; field of view 25.6 mm), a T2-weighted fast spin echo (axial section; repetition time/echo time 5462/85 milliseconds; slice thickness 4.0 mm; frequency and phase encoding matrix 512 × 256; field of view 24 mm), and a T2-weighted fluid attenuated inversion recovery (axial section; repetition time/echo time/inversion time 9500/100/2250 milliseconds; slice thickness 4.0 mm; frequency and phase-encoding matrix 512 × 256) sequences. MRI was performed in all patients to exclude the presence of widespread vascular lesions or neoplasia.

### 2.4 Dopamine imaging

DaTscan images were acquired with an INFINIA GE Hawkeye (Milwaukee, WI, United States) without scatter and attenuation correction and reconstructed using the OSEM algorithm (two iterations, 10 subsets), as previously described (10). According to the visual inspection performed by an expert Nuclear Medicine physician and a movement disorder specialist, who were blind to the patient’s diagnoses, the DaTscan was classified as either normal or abnormal.

### 2.5 Statistical analysis

Demographic, clinical and electrophysiological were compared between RT patients with and without striatal dopaminergic deficit using Fisher’s exact test for categorical variables and the Wilcoxon rank sum test or t-test for continuous variables, depending on data normality. Sensitivity, specificity, and balanced accuracy were calculated to evaluate the RT-ring’s performance in distinguishing patients with abnormal DaTscan results from those with a normal dopaminergic system, as described in Supplementary materials. Confidence intervals estimated using the exact binomial method. All statistical tests were two tailed, and the *α* level was set at *p* < 0.05. The statistical analyses were performed using R statistical software (version 4.3.2, R Foundation for Statistical Computing, 2023).

## 3 Results

### 3.1 Patients

Seventy-one patients with RT were consecutively enrolled. No patients showed MRI abnormalities consistent with exclusion criteria. Four patients were excluded from the analysis because they refused the DaTscan examination. Thus, the final cohort consisted of 67 patients (Supplementary Figure S1), including 42 patients (62.7%) with striatal dopaminergic deficit and 25 patients (37.3%) with normal DaTscan. RT patients with striatal dopaminergic deficit were slightly older and had shorter disease duration compared to those with normal DaTscan, but the two groups had a similar RT duration (Table 1).

**Table 1:** Demographic, clinical and electrophysiological data of patients with rest tremor.

Data	All RT patients (*n* = 67)	RT patients with abnormal DaTscan (*n* = 42)	RT patients with normal DaTscan (*n* = 25)	*p* value
Sex (M/F)	36 / 31	26 / 16	10 / 15	0.13^a^
Age at examination, years^b^	66.8 ± 10.1	69.2 ± 9.6	62.4 ± 9.8	**<0.05** ^**c**^
Disease duration, years^b^	7.2 ± 7.2	5.3 ± 4.2	11.7 ± 10.4	**<0.05** ^**c**^
RT duration, years^b^	4.7 ± 3.8	4.4 ± 3.5	5.4 ± 4.5	0.49^c^
Clinical MAS (Right / Left)	35/32	24 / 18	11 / 14	0.32^a^
Medication status (D/ND/N)	19/29/19	19 / 13 / 10	0 / 16 / 9	**<0.001** ^**a**^
MDS-UPDRS pars III score ^b^	21.7 ± 10.9	23.2 ± 11.3	19.2 ± 10.1	0.16^c^
MDS-UPDRS tremor score^b^	9.2 ± 6.2	8.6 ± 5.3	10 ± 7.5	0.94^c^
MDS-UPDRS rest tremor score^b^	5.2 ± 2.1	4.3 ± 2.2	3.6 ± 1.8	0.16^c^
MDS-UPDRS kinetic tremor score^b^	1.3 ± 1.2	1.1 ± 1.1	1.7 ± 1.5	0.13^c^
FTM^b^	9.9 ± 6.7	9.5 ± 5.7	10.8 ± 8.2	0.97^c^
sEMG electrophysiological data				
RT amplitude (uV)^b^	183.1 ± 146.2	245.7 ± 140.1	78.0 ± 83.0	**<0.001** ^**c**^
RT frequency (Hz)^b^	5.2 ± 0.8	4.9 ± 0.6	5.5 ± 1.0	**<0.05** ^**c**^

RT, rest tremor; MAS, most affected side; MDS-UPDRS, Movement Disorders Society-Unified Parkinson Disease Rating Scale; sEMG, surface electromyography; FTM, Fahn-Tolosa-Marin tremor rating score. D/ND/N, dopaminergic therapy/non-dopaminergic therapy/drug naïve. The MDS-UPDRS tremor score was calculated as the sum of 11 items that address tremor (items 2.10 and 3.15–3.18); the MDS-UPDRS rest tremor score was calculated as the sum of 2 items that address rest tremor (items 3.17–3.18); the MDS-UPDRS kinetic tremor score was the items 3.15. Electrophysiological data were obtained from surface electromyographic tremor analysis. All statistical tests were two tailed, and the α level was set at *p* < 0.05. The patient group with abnormal DaTscan included 40 patients with Parkinson’s Disease (PD) diagnosis, one with ET-PD syndrome and one with probable Progressive Supranuclear Palsy-Parkinsonism (PSP-P) diagnosis. The patient group with normal DaTscan included 21 patients with Essential Tremor (ET) plus, 2 patients with drug-induced tremor (1 with risperidone and 1 with valproate), and 2 patients with functional tremor. ^a^Fisher’s exact test. ^b^Data expressed as mean ± standard deviation. ^c^Wilcoxon rank sum test.

### 3.2 RT-ring report

The RT-ring mobile app generates a report showing the tremor frequency and the pattern estimation (“alternating” or “synchronous,” with the corresponding probability provided by the machine learning model) for each segment that passed the quality check (Figure 1D). A box in the upper part of the report provides information on the predominant RT pattern in that patient, based on the agreement of the estimated pattern across at least 4 out of 5 tremor recording segments, and information on the predicted DaTscan result based on the assumption that an alternating RT pattern is highly suggestive of abnormal DaTscan, while a synchronous one typically suggests a non-parkinsonian tremor. The mean time of RT ring assessment was 5.87 ± 2.87 min, allowing a quick RT assessment also during a consultation in outpatient settings.

### 3.3 RT-ring performances

The RT-ring identified a clear tremor pattern (probability of estimated pattern >70%) stable across at least 4 out of 5 tremor segments within three attempts in 62/67 patients (92.5% of the study population) (Supplementary Figure S1). The remaining five patients required more than three RT-ring sessions to obtain the report; thus, they were excluded from the main analysis, considering it is unlikely for a physician using the RT-ring to make more than three attempts in an ambulatory clinical setting. The RT-ring pattern estimation showed 96.8% agreement with the gold-standard sEMG classification (RT-ring correctly identified 36/38 patients with sEMG alternating pattern and 24/24 patients with sEMG synchronous pattern). Regarding the DaTscan prediction based on these data, the RT-ring showed 85.0% sensitivity (95% CI: 70.2–94.3%), 90.9% specificity (95% CI: 70.8–98.9%), 87.0% accuracy (95% CI: 76.1–94.3%) and 87.9% balanced accuracy in predicting the DaTscan result (Supplementary Table S1). To investigate reproducibility, the RT-ring assessment was repeated twice on the same day in 10 patients, showing the same result on DaTscan prediction.

## 4 Discussion

This study demonstrated that a small wearable device accurately predicted the DaTscan result at the individual level in RT patients, based on tremor inertial features.

There is great interest in wearable mobile digital health technologies in several branches of medicine, having these devices the potential to change how diseases are diagnosed, observed and managed, toward a more personalized and patient-centered medical approach (21). In the tremor field, a plethora of medical devices have been developed so far (22, 23); previous efforts mainly focused on detecting tremor presence, developing home monitoring systems to assess symptom severity and drug efficacy. Although there is a growing demand for tools capable of performing differential diagnosis, relatively few studies have focused explicitly on distinguishing Parkinson’s disease from essential tremor (24–32). Moreover, prior research efforts focused on tremor in general, often merging rest and action tremor data or comparing postural tremor in ET with rest tremor in PD, which are different by definition based on the circumstances when the tremor occurs (24–30). Furthermore, most previous study in the field presented results with good potential in terms of performance, but had the drawback of complex methodology requiring extensive data processing, thus making previous devices mainly suitable for research settings (24–30). On the other hand, the RT-Ring is a small ring-shaped mobile device with a user-friendly mobile app which has the potential to help clinicians in the differential diagnosis between parkinsonian and non-parkinsonian RT syndromes (i.e., ET plus), it has been patented (patent no: 102021000019793) and is conform to European safety regulations, on track for a broad distribution. The RT-Ring is based on machine learning technology using accessible RT inertial features to estimate the muscular contraction RT pattern, a feature which has proven to accurately predict DaTscan result (7–11). This model employs random forest algorithm and uses as input tremor features including amplitude, coherence, harmonics, frequency and other tremor characteristics which are automatically extracted from the inertial tremor signals recorded by the device. The rationale of developing such a device to predict the tremor pattern lies in its ability to distinguish patients with and without striatal dopaminergic deficit (10). Indeed, in a previous study on a large cohort of more than 200 RT patients, we demonstrated that RT pattern significantly outperformed other canonical tremor features such as RT amplitude or frequency in this diagnostic task (10), providing a solid basis for the current study. Moreover, RT pattern has the advantage to be a very stable feature over time (10), while tremor amplitude can fluctuate due to factors such as stress, mental concentration and medications, reducing its reliability for the differential diagnosis of rest tremor. The RT-Ring is a simple user-friendly wearable device allowing an easy and fully automated evaluation of the RT pattern and also overcomes the current limitations of pattern sEMG assessment, representing a further step forward in the field. In fact, unlike sEMG, which demands expensive equipment and expertise for electrode placement, and relies on pattern visual subjective interpretation, the RT-ring does not require any specific expertise and allows automated operator-independent pattern estimation based on robust machine learning technology.

A strength of this study is its prospective design, with all patients undergoing RT-ring assessment, sEMG tremor analysis, and DaTscan in addition to clinical examination. A limitation is the relatively limited sample size, which highlights the need for larger multicenter studies to validate the findings and investigate the benefits for health outcomes and the healthcare economic burden.

In conclusion, the RT-ring is a promising non-invasive wearable mobile device for the differential diagnosis of rest tremor disorders. It can be used by general practitioners and physicians with no expertise in tremor, making it suitable as an inclusive screening tool for primary care and outpatient settings. This strategy may potentially improve the referral of RT patients to neurology, reducing the diagnostic delay and supporting the tremulous PD clinical diagnosis in settings with limited access to dopamine imaging. It may also mitigate diagnostic disparities across populations related to social conditions and limited-income regions.

## Data Availability

The raw data supporting the conclusions of this article will be made available by the authors, without undue reservation.

## Supplementary material

Supplementary Figure S1 The figure shows a flowchart of the study inclusion/exclusion procedures. Among the five patients requiring more than 3 RT-ring sessions, 3 patients required 4 sessions, 1 patient required 5 sessions, and 1 patient did not show a stable RT pattern within 5 sessions (maximum number of attempts). RT, rest tremor.
