# Corrigendum: RT-ring: a small wearable device for tremulous Parkinson's disease diagnosis in primary care

**DOI:** 10.3389/fneur.2025.1588171

**Published:** 2025-03-20

**Authors:** Jolanda Buonocore, Basilio Vescio, Marida De Maria, Marianna Crasà, Rita Nisticò, Pier P. Arcuri, Giuseppe L. Cascini, Anna Latorre, Aldo Quattrone, Andrea Quattrone

**Affiliations:** ^1^Institute of Neurology, Department of Medical and Surgical Sciences, Magna Graecia University, Catanzaro, Italy; ^2^Neuroscience Research Center, Magna Graecia University, Catanzaro, Italy; ^3^Biotecnomed S.c.ar.l., Catanzaro, Italy; ^4^Institute of Bioimaging and Complex Biological Systems, IBSBC-CNR, Catanzaro, Italy; ^5^Institute of Radiology, Azienda Ospedaliero-Universitaria Renato Dulbecco, Catanzaro, Italy; ^6^Nuclear Medicine Unit, Azienda Ospedaliera-Universitaria Dulbecco, Catanzaro, Italy; ^7^Department of Experimental and Clinical Medicine, Magna Graecia University, Catanzaro, Italy; ^8^Department of Clinical and Movement Neurosciences, UCL Queen Square Institute of Neurology, University College London, London, United Kingdom

**Keywords:** rest tremor, tremor pattern, wearable device, RT-ring, DaTscan, Parkinson's disease, essential tremor plus, machine learning

In the published article, there was an error regarding the affiliation(s) for Basilio Vescio. As well as having affiliation 3 Biotecnomed S.C.ar.l., Catanzaro, Italy, they should also have the affiliation 4 Institute of Bioimaging and Complex Biological Systems, IBSBC-CNR, Catanzaro, Italy.

The authors apologize for this error and state that this does not change the scientific conclusions of the article in any way. The original article has been updated.

