# Supplementary figures and images for: RT-ring: a small wearable device for tremulous Parkinson’s disease diagnosis in primary care
Source: Front Neurol. 2025 Jan 27;16:1534205. doi: 10.3389/fneur.2025.1534205 (PMC11807809; doi:10.3389/fneur.2025.1534205)

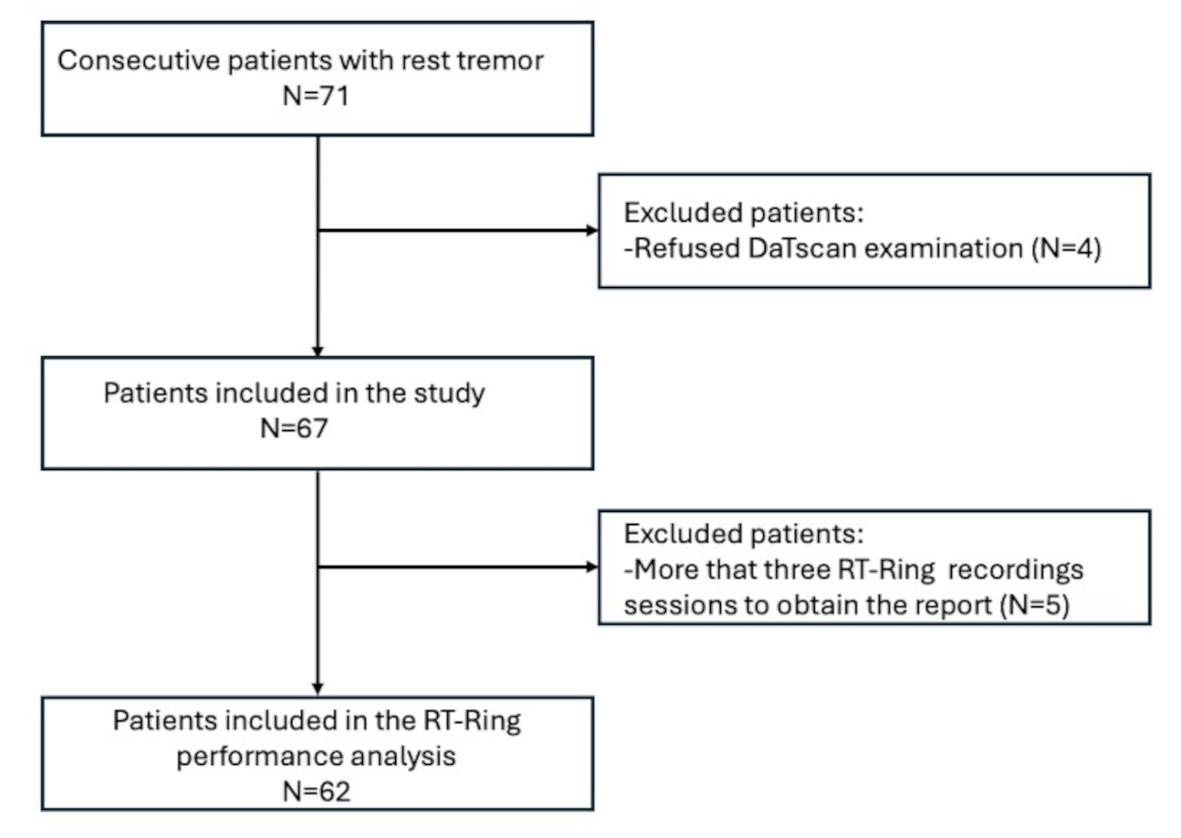

Supplement: Supplementary Figure S1 — The figure shows a flowchart of the study inclusion/exclusion procedures. Among the five patients requiring more than 3 RT-ring sessions, 3 patients required 4 sessions, 1 patient required 5 sessions, and 1 patient did not show a stable RT pattern within 5 sessions (maximum number of attempts). RT, rest tremor. [file Image_1.jpeg]
