# Supplementary material for: RT-ring: a small wearable device for tremulous Parkinson’s disease diagnosis in primary care
Source: Front Neurol. 2025 Jan 27;16:1534205. doi: 10.3389/fneur.2025.1534205 (PMC11807809; doi:10.3389/fneur.2025.1534205)
Supplement: Supplementary file 2 [file Data_Sheet_1.docx]

**Supplementary Material**

**Methods**

**RT-Ring tremor data acquisition and preprocessing workflow**

1. Data acquisition and preprocessing

- Tremor data acquisition:
  - Hand positioning is verified before recording to ensure accurate data acquisition and minimize postural tremor. The hand is positioned at rest, typically hanging from the chair armrest.
  - The RT-ring records inertial data (acceleration and angular velocity) from a finger of the tremulous hand using a triaxial accelerometer and gyroscope. Five distinct segments lasting 10 seconds each are recorded. For each segment, the data are transmitted via Bluetooth Low Energy to a smartphone app in real time, which provides graphical guidance to the user through the whole process. The data are transferred to a remote server for the following processing.
- Signal filtering and quality control:
  - Raw signals are filtered to remove artifacts and noise and segmented into 10-second windows.
  - Each segment undergoes a quality check to confirm the presence of tremor, defined as a rhythmic movement, with frequency falling within the 2–10 Hz range and that remains stable over time.
  - Segments failing quality check are discarded and the recording is repeated.

2. Tremor feature extraction

- The RT-ring inertial features include:
  - Spectral analysis (Fourier Transform): tremor frequency and spectral amplitude.
  - Wavelet Transform: decomposition of the signal to extract detail and approximation coefficients.
  - Statistical features: mean, variance, skewness, and kurtosis of spectral coherence and cross-spectral components.
  - Single-axis analysis: frequencies and amplitudes for each accelerometer and gyroscope axis.
  - Peak-to-peak amplitudes from accelerometer and gyroscope data.
  - Harmonic components are extracted and summed to aid in tremor classification.

Comprehensive data on feature extraction are available in a previous publication [1].

3. Machine Learning model:

- A previously developed random forest (RF) model based on inertial features was selected for tremor pattern classification, frozen after a previous study [1] and applied without modification in the current study. The implemented model is a binary classifier to estimate the tremor electromyographic pattern with two possible outcomes, representing:
  - Rest tremor with inertial features suggestive of synchronous tremor pattern (meaning antagonist muscle contraction burst occurring at the same time).
  - Rest tremor with inertial features suggestive of alternating tremor pattern (meaning shifted contraction burst).

The trained machine learning model was deployed on a remote server using Python (Flask framework). Tremor classification results (alternating or synchronous pattern) are transmitted to the smartphone app within 5 seconds of data acquisition

4. Final report

- The mobile app guides the physician through the acquisition of five tremor segments. Each of them undergoes all the processing steps described above.
- If at least 4 out of 5 segments display a consistent pattern with high prediction probability (≥70%), the tremor is classified as alternating (typically associated with an abnormal DaTscan result) or synchronous (typically associated with a normal DaTscan result). If consistency is not achieved, the recording session is repeated.
- The app generates a report for visual interpretation of the results, indicating the predominant estimated tremor pattern and the prediction on DaTscan result.

**Classification Performance of RT-ring in distinguishing patients with abnormal DaTscan from those with normal dopaminergic system**

Sensitivity, specificity, and accuracy of RT-ring in distinguishing patients with abnormal DaTscan results from those with normal dopaminergic function were calculated by directly comparing RT-ring predictions with DaTscan outcomes, as follows:

*Sensitivity = TP / (TP + FN)*

*Specificity = TN / (TN + FP)*

*Accuracy = (TP + TN) / (TP + TN + FP + FN)*

*Balanced accuracy = (sensitivity + specificity) / 2*

Where True positives (TP) were cases where both the RT-ring and DaTscan indicated a positive result.

False positives (FP) were cases where the RT-ring predicted a positive Datscan result but the examination turned out to be normal.

True negatives (TN) were cases where both the RT-ring and DaTscan indicated a negative result.

False negatives (FN) were cases where the RT-ring predicted a negative Datscan result but the examination turned out to be abnormal.

**Supplementary Material’s References:**

1. Vescio B, De Maria M, Crasà M, et al. Development of a New Wearable Device for the Characterization of Hand Tremor. Bioengineering (Basel). 2023; 10(9):1025. doi: 10.3390/bioengineering10091025.
