# Supplementary material for: RT-ring: a small wearable device for tremulous Parkinson’s disease diagnosis in primary care
Source: Front Neurol. 2025 Jan 27;16:1534205. doi: 10.3389/fneur.2025.1534205 (PMC11807809; doi:10.3389/fneur.2025.1534205)
Supplement: Supplementary file 3 [file Table_1.docx]

**Supplementary Table 1:** RT-ring classification performance in distinguishing rest tremor patients with striatal dopaminergic deficit from those with normal DaTscan.

| **RT-ring performance** | **Patient**  **Number** | **Classification**  **performance** | **95% Confidence intervals** |
| --- | --- | --- | --- |
| sensitivity | 34/40 | 85.0% | 70.2% – 94.3% |
| specificity | 20/22 | 90.9% | 70.8% – 98.9% |
| Accuracy | 54/62 | 87.0% | 76.1% – 94.3% |

Abbreviations: RT = rest tremor. RT patients with dopaminergic deficit were considered as positive class.
